# Shingles-Like Cutaneous Manifestation of Herpes Simplex Virus Type 1 in a Young Male Patient: A Case Report

**DOI:** 10.7759/cureus.107034

**Published:** 2026-04-14

**Authors:** John Acosta-Peñaloza, Osman Mahboob, Jane Hufnagel, Yusuf Amawi, Cynthia Tie

**Affiliations:** 1 Clinical Sciences, Florida State University College of Medicine, Tallahassee, USA; 2 Dermatology, Family Dermatology of North Florida, Tallahassee, USA

**Keywords:** atypical dermatologic presentation, herpes simplex virus type 1, shingles mimic, valacyclovir, viral rash

## Abstract

Herpes simplex virus type 1 (HSV-1) is a common DNA virus that typically causes orolabial lesions. Atypical presentations, such as dermatomal vesicular eruptions, are rare and may mimic varicella-zoster virus (VZV) reactivation, complicating diagnosis. Here we present a case of HSV-1 with recurrent vesicular eruptions in the C4-C5 dermatomes, initially posited to be herpes zoster. Viral culture confirmed HSV-1, and the patient’s symptoms were successfully managed with valacyclovir and topical corticosteroids.

## Introduction

Herpes simplex virus type 1 (HSV-1) is a double-stranded DNA virus frequently implicated in the development of orolabial lesions [1]. Transmission primarily occurs through direct contact with infected bodily fluids or lesions, establishing a lifelong latent infection [2]. Acquisition typically involves interaction with the buccal or labial mucosa, often via close personal contact [3]. Epidemiological data estimate that HSV-1 affects approximately 67% of the global population under the age of 50 [4].

HSV-1 may occasionally present with recurrent vesicular eruptions in localized dermatomal patterns, an atypical manifestation that can mimic varicella-zoster virus (VZV) reactivation and complicate clinical evaluation [5]. These cases reflect a complex interplay between viral reactivation, host immune response, and individual patient factors, contributing to variability in presentation [6]. HSV-1 may rarely reactivate along non-trigeminal sensory pathways, potentially due to latency within dorsal root ganglia and localized immune or stress-related triggers, resulting in atypical dermatomal distributions. Accurate diagnosis, therefore, requires a comprehensive approach, including a thorough history, detailed physical examination, and confirmatory diagnostic tools such as Tzanck smear, polymerase chain reaction (PCR), or viral cultures [7].

This report aims to highlight the diagnostic challenge of dermatomal HSV-1 mimicking herpes zoster and to emphasize the importance of confirmatory testing in recurrent or atypical cases.

In this report, we present a case of HSV-1 infection with an atypical presentation characterized by vesicular eruptions in a dermatomal distribution. We emphasize the diagnostic and therapeutic considerations involved in managing such uncommon manifestations to improve clinical recognition and patient outcomes.

## Case presentation

A 19-year-old male patient with no significant medical history presented with a localized rash on the posterior left shoulder. The patient reported pruritus the day before presentation, but denied itching at the time of evaluation. No other areas were affected. The patient denied recent medication use, preceding infections, new topical products, travel history, insect bites, new or multiple sexual partners, and contact with individuals with similar eruptions. He did report a similar rash in the same location eight years prior, which resolved spontaneously. There was no personal or family history of dermatological conditions and no known allergies. The patient denied alcohol and drug use and was a student by occupation. Systemic symptoms such as cough, fever, sore throat, chills, diarrhea, and joint aches were absent.

**Figure 1:**
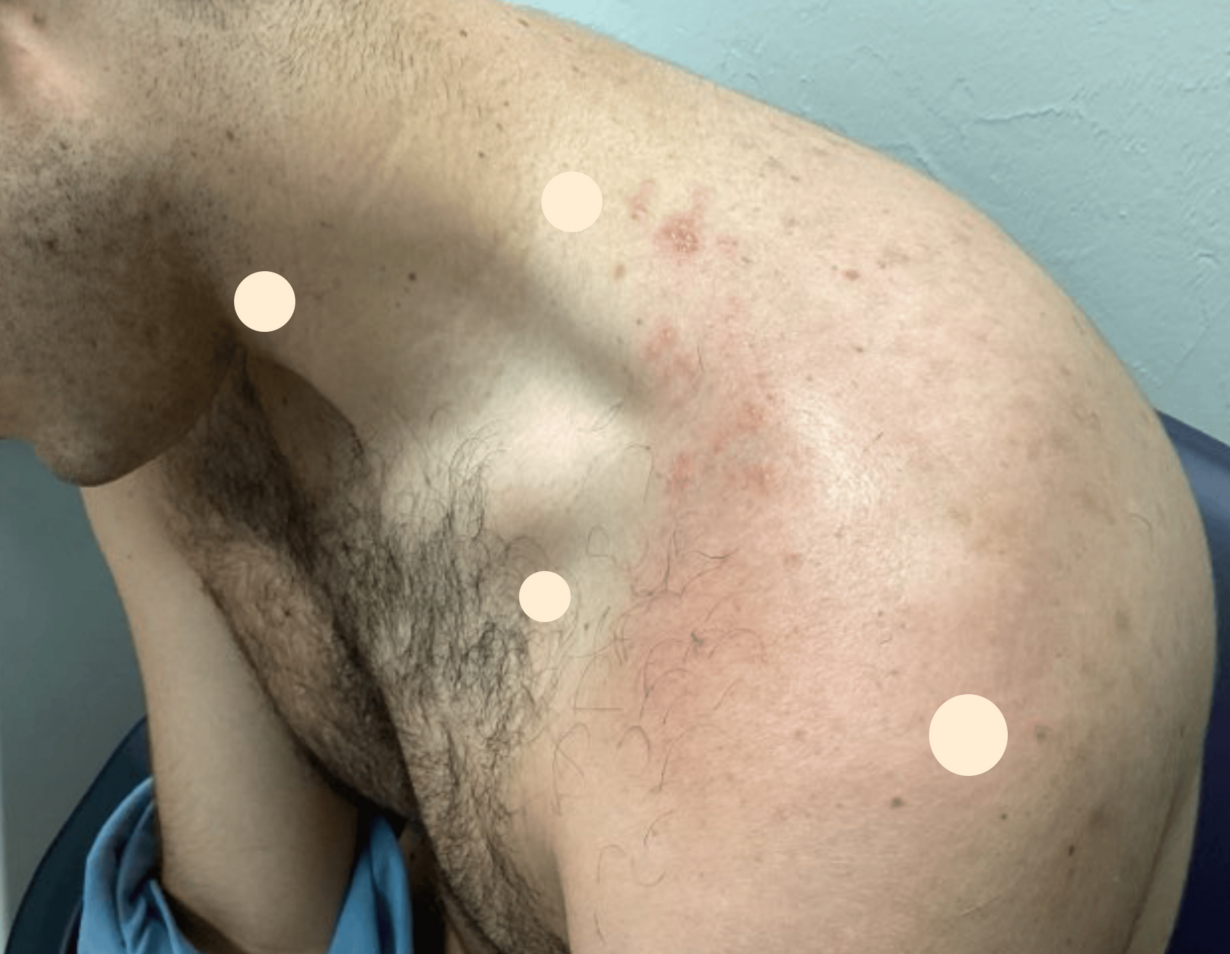
Patient's left anterolateral shoulder displaying grouped vesicles on a urticarial pink plaque in a dermatomal pattern

On examination, grouped vesicles on a urticarial pink plaque were noted over the posterior left shoulder, corresponding to the C4 and C5 dermatomes (Figure 1).

A Tzanck smear demonstrated multinucleated giant cells. Because of the patient’s dermatomal distribution, pruritic symptoms, and smear findings, a diagnosis of herpes zoster was supported. Initial management included valacyclovir 1 g orally three times daily for seven days, and topical triamcinolone ointment applied twice daily, with instructions for follow-up as needed. The lesions progressed from vesicular eruptions to crusting over several days before complete resolution without scarring. There was no associated lymphadenopathy, sensory deficits, or surrounding neurologic changes.

Four years later, the patient returned with recurrent flares of the rash occurring approximately twice per year, each episode lasting five to seven days with a similar clinical course. On examination, the same vesicular eruption was present in the original location, with onset the day prior. Valacyclovir 1 g orally three times daily for seven days was prescribed, and topical treatment was adjusted to clobetasol 0.05% cream applied twice daily.

Given the rash’s recurrence and atypical dermatomal distribution, confirmatory testing with viral culture was performed, revealing HSV-1. This finding revised the initial diagnosis.

The patient later reported that his HSV-1 symptoms remained well-controlled with the current regimen. He did not require further dermatology follow-up and noted no complications. Overall, the disease course consisted of an initial episode, an extended asymptomatic interval of eight years, followed by recurrent flares occurring approximately twice annually prior to definitive diagnosis.

## Discussion

Herpes zoster and HSV-1 are viruses of the herpesviridae family that remain latent in dorsal root ganglia following primary infection [8,9]. VZV, the causative agent of herpes zoster, reactivates when triggered by immunosuppression or stress. This causes viral replication and retrograde transport along corresponding sensory nerves, resulting in a painful, dermatomal vesicular rash [8]. HSV-1 reactivates due to physical or emotional stress, and can present in a variety of forms depending on the initiation site, the host’s immune state, and whether the infection is primary or recurrent. The most common presentations manifest in the orofacial region, as the main site of latency is the sensory trigeminal ganglion [9]. However, in rare cases, HSV-1 reactivation can manifest as a vesicular rash in non-facial, atypical dermatomes, mimicking VZV presentations [5]. Both conditions can present with grouped vesicles on an erythematous base with associated pruritus and pain, and multinucleated giant cells on Tzanck smear. A viral culture or polymerase chain reaction (PCR) is therefore needed to confirm a diagnosis. 

In the presented case, initial findings of a localized dermatomal rash with vesicles on the posterior left shoulder (C4-C5 distribution) supported a diagnosis of herpes zoster. However, recurrent flares and viral culture confirmed by monoclonal antibody microscopic immunofluorescence contributed to the final diagnosis of this unusual presentation of HSV-1. 

HSV-1 has global seroprevalence rates exceeding 50% by adolescence, with higher rates observed in developing countries. HSV-1 affects approximately 67% of the global population under the age of 50, according to epidemiologic data [4]. It is typically seen causing orolabial lesions, with its presence in non-traditional dermatomes being infrequent. Case reports have emphasized the diagnostic challenges associated with identifying these various types of atypical presentations of HSV-1 infection, as these case presentations are relatively rare and often underreported [10]. Reactivation rates vary based on factors such as immune status, trauma, or stress. In contrast, VZV occurs predominantly in older or immunocompromised individuals, with incidence rates sharply rising after age 50. It affects approximately 30% of the population at least once in their lifetime, with an annual incidence estimated at 3.2 cases per 1,000 in the general population. This number increases to an average of 9.1 cases per 1,000 among those over 60 years of age [11]. 

A thorough patient history and physical examination remain critical in differentiating between HSV-1 and VZV. Laboratory tests, including Tzanck smear, PCR, and viral culture, are essential tools for accurate diagnosis, particularly in atypical presentations where clinical features overlap [12]. Viral culture serves as a definitive diagnostic modality in distinguishing HSV-1 from VZV in complex or recurrent cases, as demonstrated in this patient.

Early treatment with valacyclovir and topical corticosteroids when managing HSV-1 can provide comfort and relief of symptoms. Valacyclovir’s high bioavailability and efficacy in reducing viral replication make it a cornerstone of acute and prophylactic management for HSV-1. Its proven ability to control recurrent flares ensures effective long-term suppression of reactivation events, particularly in patients prone to frequent recurrences [13]. Valacyclovir was selected due to its high oral bioavailability and established efficacy in reducing HSV-1 viral replication and recurrence frequency. The addition of topical corticosteroids was based on evidence demonstrating improved symptom control and reduced lesion duration when combined with antiviral therapy.

Topical corticosteroids, when combined with antiviral therapy such as valacyclovir, have been shown to significantly reduce the recurrence of ulcerative lesions in recurrent herpes labialis compared to antiviral therapy alone. This combined approach also decreases the duration of illness, providing enhanced patient comfort during outbreaks, without a significant increase in adverse effects [14]. Tailored treatment plans, incorporating valacyclovir and addressing individual patient factors, ensure the most effective management of HSV-1.

Atypical presentations of HSV-1 require tailored antiviral regimens based on recurrence patterns and patient response. Incorporating valacyclovir as part of individualized treatment strategies is essential for managing these rare cases. Additionally, patient education on recognizing early symptoms and adherence to antiviral prophylaxis is key in preventing recurrent flares.

Atypical presentations of HSV-1, such as the case described, highlight the importance of thorough diagnostic evaluation. The overlapping clinical features of HSV-1 and VZV, i.e., vesicular rashes, dermatomal distribution, and associated pain, can lead to diagnostic uncertainty. While clinical examination and history are essential, confirmatory tests such as viral culture or PCR are critical for accurate identification, particularly in recurrent or treatment-resistant cases.

From a management perspective, recognizing atypical HSV-1 presentations ensures the timely initiation of antiviral therapy, potentially preventing complications and reducing symptom duration. Additionally, reporting such cases contributes to a deeper understanding of HSV-1’s clinical spectrum and informs future diagnostic and therapeutic strategies.

## Conclusions

This case highlights the importance of considering HSV-1 in atypical dermatomal eruptions, particularly when clinical features overlap with herpes zoster. While based on a single patient, this case emphasizes the value of confirmatory diagnostic testing in recurrent or atypical presentations to guide appropriate management.
